# Metformin Suppresses Hepatocellular Carcinoma through Regulating Alternative Splicing of LGR4

**DOI:** 10.1155/2022/1774095

**Published:** 2022-11-04

**Authors:** Han Zhuo, Shuying Miao, Zhenquan Jin, Deming Zhu, Zhenggang Xu, Dongwei Sun, Jie Ji, Zhongming Tan

**Affiliations:** ^1^Hepatobiliary Center, The First Affiliated Hospital of Nanjing Medical University, Nanjing, Jiangsu, China; ^2^Department of Pathology, Nanjing Drum Tower Hospital, The Affiliated Hospital of Nanjing University Medical School, Nanjing, Jiangsu, China; ^3^The First Clinical Medical College of Nanjing Medical University, Nanjing, Jiangsu, China; ^4^Jiangsu Breast Disease Center, The First Affiliated Hospital of Nanjing Medical University, Nanjing, Jiangsu, China

## Abstract

**Methods:**

First, the expression of LGR4 in HCC tumor tissues and cell lines was detected by western blotting and immunofluorescence. The ability of cell proliferation, migration, and invasion was detected with CCK8, wound-healing, and transwell assays when overexpressing LGR4 or treating with metformin. The *β*-catenin expression was detected by immunofluorescence. In order to investigate novel AS-associated LGR4, we discarded LGR4 isoforms from GSO databases. We used siRNA to knock down the specific isoform to check the cell proliferation, migration, and invasion when treated with metformin.

**Results:**

The level of LGR4 expression was higher in HCC cell lines and tumor tissues. The HCC cell proliferation, migration, and invasion were increased when overexpressing LGR4, which could be reduced by metformin treatment. The GEO database (GSE190076) showed that LGR4 had switching properties in HCC cell lines treated with metformin. We used siRNA to knock down the specific isoform, and the result showed that the specific isoform siRNA could promote the inhibition of cell invasion caused by metformin treatment.

**Conclusions:**

LGR4 could promote the ability of cell proliferation, migration, and invasion in HCC, which could be reduced by metformin through alternative splicing.

## 1. Background

Hepatocellular carcinoma is the most common type of liver cancer all over the world, especially in China [1, 2], although the treatment is still developing and includes surgery, transplantation, and local therapies. Drug therapy is also applied in clinics [2, 3]. However, the mechanism of HCC is still unclear. Metformin is a dimethyl biguanide to treat type 2 diabetes mellitus (T2DM) with few side effects. Metformin can be used to treat many types of cancer, including colorectal [4] and prostate cancers [5]. In HCC, metformin could regulate the expression of FOXO3 by apoptosis and pyroptosis to inhibit the development of hepatocellular carcinoma [6].

LGR4 belongs to the GPCRs superfamily and is recognized as a trans-membrane receptor of the LGR family. Recently, a study indicated that LGR4 could interact with PrPc to promote tumorigenesis and liver metastasis by stemness of colorectal cancer stem cells [7]. In acute myeloid leukemia, RSPO3-LGR4 signaling could be recognized as a target for treatment [8]. It also can be related to poor prognosis in ovarian cancer [9]. However, the function of LGR4 is still unclear. Alternative splicing could produce multiple transcripts of mRNA to regulate the gene expression. An MTA1 splicing switch can be regulated by RALY to activate the cholesterol-related pathway in hepatocellular carcinoma [10]. The alternative splicing could also be regulated by DDX17 and produce a PXN-AS1 isoform in HCC. However, whether metformin could regulate gene alternative splicing is still unclear.

In our research, we investigated the function of LGR4 in the HCC, and we also found that metformin could reduce the expression of LGR4. We also indicated the mechanism of the regulatory effect of metformin. The study could provide evidence of LGR4 function, which could be recognized as a target for HCC treatment.

## 2. Methods

### 2.1. Cell Culture and Treatment

Normal cell lines (L02) and Huh-7 and HepG3B cell lines were obtained from the American Type Culture Collection (ATCC). All cells were cultured in DMEM (Invitrogen, USA) with FBS (10%), in an atmosphere containing 5% CO_2_ at 37°C. The HCC cell lines were treated with metformin (20 *μ*M) [6]. The HCC cell lines were transfected with PcDNA-LGR4 (Genewiz, China) and siRNA-LGR4 (GenePharma, China) by using Lipo 3000 (Thermo, USA). siRNA-LGR4 sequence: UGGAUGCCGCUCAUCCUAAAG.

### 2.2. CCK-8 Assay

According to the previous procedure [11], the treated cells were added into wells with a proper density. Two days later, the cell counting kit-8 (CCK-8) reagent was added to the wells for 2 hours. We determined the optical density (OD) at 450 nm using a multimode microplate reader.

### 2.3. Cell Invasion Assay

The treated cells were digested and added into the top chambers of transwell inserts with FBS-free DMEM. The cell density is 2 × 10^5^ cells per well. And DMEM (10% FBS) was cultured in the bottom chambers. After 6 hours, 4% paraformaldehyde was used to fix the inserts and stain them with 0.1% crystal violet solution. Also, the images were obtained under a microscope.

### 2.4. Wound Healing Assay

The treated cells were digested and cultured in 6-well plates. After treatment, a 200 *μ*L pipette tip was used to scratch, cells were washed, and the cell was incubated with DMEM for another 12 hours. Three random areas were photographed to assess the distance of migration.

### 2.5. Western Blotting

According to the previous procedure [11], proteins were isolated in RIPA buffer containing phosphatase and protease inhibitors (Roche, US). Equal total proteins were separated by SDS/PAGE gels to blot onto PVDF membranes (Millipore, USA). After blocking, the bands were incubated with an anti- LGR4 antibody (ab75501; Abcam). Finally, the blot was observed via the ECL detection system.

### 2.6. Immunofluorescence Assay

For *β*-catenin staining, the treated cells were washed with PBS three times and fixed, then treated with 0.1% Triton X (Beyotime, China). Subsequently, a blocking buffer was added and the primary antibody was incubated overnight at 4°C. The next day, the treated cells were incubated with a secondary antibody, then treated with DAPI. Finally, the images were captured by a microscope.

### 2.7. Bioinformatic Analysis

The GEO database (GSE190076) was used to indicate the different gene expressions. Then, the transcript was analyzed with an isoform switch analyser. A total of 480 genes underwent isoform switching. KEGG pathway analysis was also used to analyze the genes' function.

### 2.8. Statistical Analysis

All measurements were presented as the mean ± standard deviations (SD) from three independent experiments. Statistical significance was defined as a *p* value < 0.05. Differences were determined using a two-way analysis of variance (ANOVA) or unpaired Student's *t*-test by Prism software.

## 3. Results

### 3.1. LGR4 was Increased in HCC Tissues and Cell Lines

**Figure 1:**
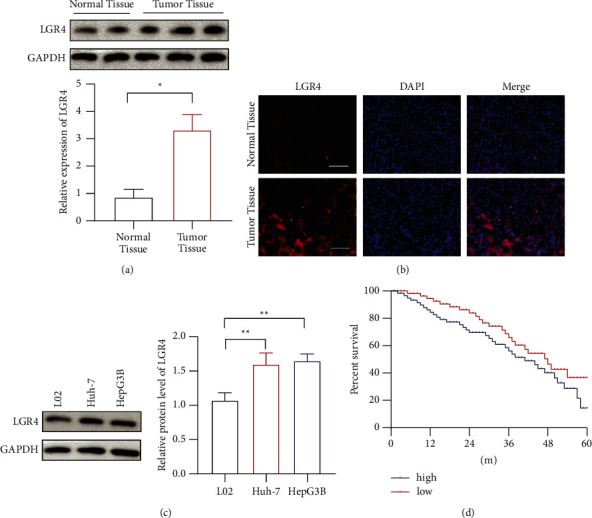
The level of LGR4 expression in HCC. (a)-(b). The level of LGR4 expression in HCC tumor tissues by western blotting and immunofluorescence. (c). The level of LGR4 expression in HCC cell lines. (d). Multivariate Cox analysis revealed that LGR4 in HCC tissues was an independent prognostic factor in HCC patients. ^∗^*p* < 0.05, ^∗∗^*p* < 0.01, ^∗∗∗^*p* < 0.001.

First, the level of LGR4 expression was detected in HCC tissues. It showed that LGR4 expression was increased in the tumor tissues (Figure 1(a)). We also used immunofluorescence to check the expression of LGR4, and it showed the same results (Figure 1(b)). Then, we checked the LGR4 expression in the HCC cell lines. The LGR4 expression was increased in Huh-7 and HepG3B cell lines (Figure 1(c)). It indicated that LGR4 expression was upregulated in tissues and at cell levels. Multivariate Cox analysis revealed that the expression of LGR4 in HCC tissues could be acting as an independent prognostic factor in HCC patients (Figure 1(d)).

### 3.2. LGR4 Promoted Cell Proliferation, Migration, and Invasion

**Figure 2:**
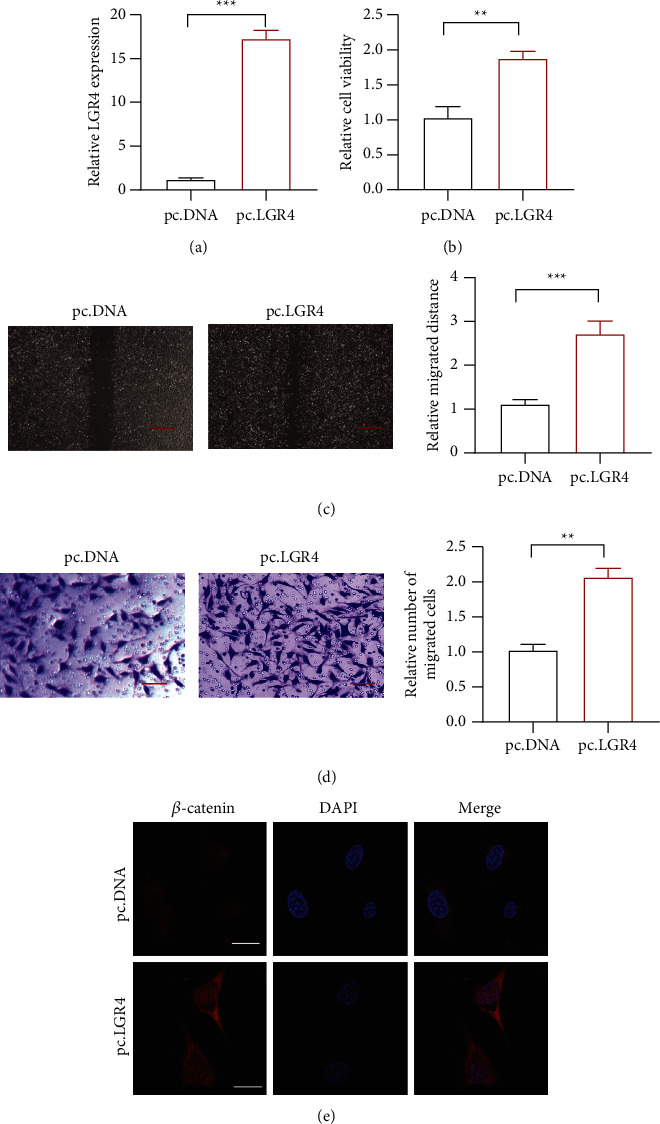
The role of LGR4 in HCC. (a) The expression of LGR4 was detected upon LGR4 overexpression. (a). The mRNA level of LGR4 in the LGR4 overexpressed cells. (b). The proliferation of HCC by LGR4 overexpression in HCC cell lines. (c). The migration of HCC by LGR4 overexpression in HCC cell lines; scar bar = 100 *μ*m. (d). The invasion of HCC by LGR4 overexpression in HCC cell lines; scar bar = 50 *μ*m. (e). The expression of catenin in LGR4 overexpressed HCC cell lines. ^∗∗^*p* < 0.01, ^∗∗∗^*p* < 0.001. PcDNA, an empty vector.

To explore the role of LGR4 in HCC cell lines, we overexpressed LGR4 to check cell proliferation, migration, and invasion in HCC cell lines. First, we used RT-qPCR to check the expression of LGR4 in overexpressed cell lines (Figure 2(a)). It demonstrated that the level of LGR4 expression was much higher, indicating the successful transfection. Then, a CCK8 assay was carried out to check the cell viability, and the result demonstrated that LGR4 could promote cell proliferation (Figure 2(b)). The wound healing assay and a transwell assay also indicated the LGR4 overexpression promoted cell migration and invasion (Figures 2(c) and 2(d)). The above result showed that LGR4 could act as an oncogene in the HCC. LGR4 could activate the wnt/*β*-catenin pathway in many cancers. So, the immunofluorescence indicated that the *β*-catenin expression was higher in the LGR4 overexpressed cells (Figure 2(e)). It indicated that the LGR4 could activate the wnt/*β*-catenin pathway in HCC.

### 3.3. Metformin Could Reduce the Cell Proliferation, Migration, and Invasion through LGR4

**Figure 3:**
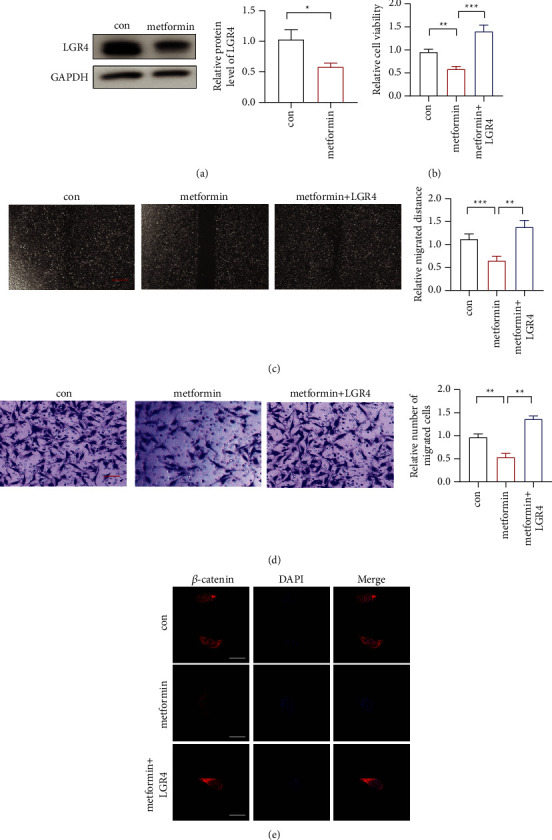
Metformin could reduce tumorigenesis through LGR4. (a). The expression of LGR4 was detected upon metformin treatment. (b). The proliferation of HCC by LGR4 overexpression in metformin-treated HCC cell lines. (c). The migration of HCC by LGR4 overexpression in metformin treated HCC cell lines; scar bar = 100 *μ*m. (d). The invasion of HCC by LGR4 overexpression in metformin-treated HCC cell lines; scar bar = 50 *μ*m. (e). The expression of catenin by LGR4 overexpression overexpressed metformin-treated HCC cell lines. ^∗∗^*p* < 0.01, ^∗∗∗^*p* < 0.001. PcDNA, an empty vector.

Metformin, an oral hypoglycemic drug, exerts anticancer effects in many cancers, especially in HCC. To explore the effect of metformin on LGR expression, we checked LGR4 expression in HCC cell lines treated with metformin. It showed that metformin could reduce the LGR4 expression (Figure 3(a)). Then, we overexpressed LGR4 when treated with metformin to check the proliferation, migration, and invasion of cells. The results showed that metformin could reduce cell proliferation, migration, and invasion, which are counteracted by LGR4 overexpression (Figures 3(b)–3(d)). Then, we detected the expression of catenin, and it showed that metformin reduced the catenin pathway through LGR4 (Figure 3(e)).

### 3.4. Metformin Decreased the LGR4 Expression by Alternative Splicing

**Figure 4:**
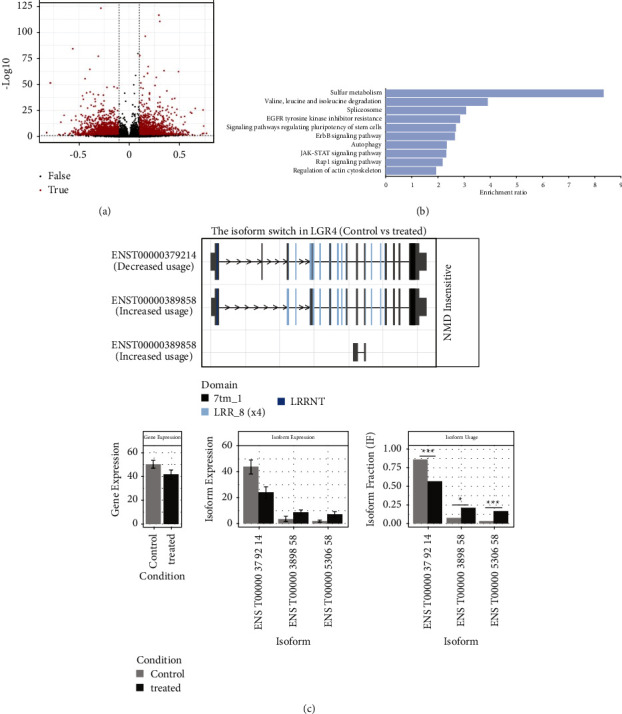
Metformin decreased the LGR4 expression by alternative splicing. (a). Volcano plots shows genes with differential transcript usage (differential isoform fraction (dIF) > 0.3 and FDR < 0.01). (b). KEGG pathways enriched for genes with differential transcript usage. (c). Visualization of switched isoform structure LGR4. Three isoforms showed differential isoform expressions, although there was no difference for the overall gene expression. Treated: metformin treatment.

To identify the mechanism by which LGR4 expression is regulated by metformin. We used the GEO database (GSE190076) to indicate the different gene expressions. It showed the mRNA level of LGR4 had no difference between the two groups, which meant it may be regulated by alternative splicing. Then, we analyzed transcript usage with an isoform switch analyser. A total of 480 genes underwent isoform switching (Figure 4(a)). KEGG pathway analysis also showed that the genes were enriched in spliceosome (Figure 4(b)). Interestingly, the transcript ENST00000379214 of the LGR4 gene decreased in HCC cell lines (Figure 4(c)). It indicated that metformin could reduce the level of LGR4 expression through alternative splicing.

### 3.5. LGR4 Switching Genes RNAi Reduce the Antitumor Effect Caused by Metformin

**Figure 5:**
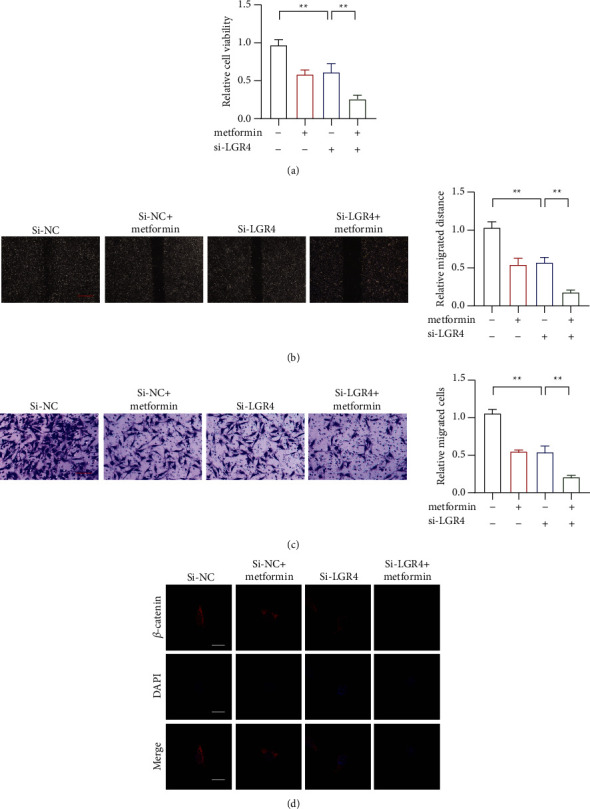
LGR4 switching genes RNAi reduces the antitumor effect caused by metformin. (a). The proliferation of HCC by LGR4 switching genes RNAi in metformin-treated HCC cell lines. (b). The migration of HCC by LGR4 switching genes RNAi in metformin-treated HCC cell lines; scar bar = 100 *μ*m. (c). The invasion of HCC by LGR4 switching genes RNAi in metformin-treated HCC cell lines; scar bar = 50 *μ*m. (d). The expression of catenin was induced by LGR4 switching genes by RNAi metformin-treated HCC cell lines. ^∗∗^*p* < 0.01, ^∗∗∗^*p* < 0.001. PcDNA, an empty vector.

Finally, we used siRNA to knock down the isoforms to observe the phenotypic change in the metformin treatment. In the CCK8 assay, it showed that metformin treatment could reduce cell proliferation, which was promoted by RNAi (Figure 5(a)). We also used the wound healing assay and the transwell assay to show that a specific isoform siRNA could promote the inhibition of cell migration and invasion caused by metformin treatment (Figures 5(b) and 5(c)). We also detect the *β*-catenin expression. It indicated that the specific isoform siRNA could also reduce the expression of catenin in response to metformin treatment (Figure 5(d)).

## 4. Discussion

Despite the application of surgery, transplantation, and local therapies in HCC treatment, the rate of death caused by HCC remains high. Recently, metformin, which is recognized to treat T2DM, was shown that to have antitumor effects. In colorectal cancer (CRC), the use of metformin may improve disease-free survival and overall survival in CRC patients with T2DM [12]. In bladder cancer cells, metformin improves the antitumor effect of Olaparib through the STAT3/C-MYC pathway [13]. In HCC, metformin could regulate the Hippo signaling pathway to reduce interleukin-22-induced hepatocellular carcinoma [14]. Metformin could also sensitize sorafenib-resistant HCC cells through autophagy by AMPK activation [15]. However, the mechanism of the antitumor effect of metformin is still to be explored.

LGR4 belongs to the GPCRs superfamily and is recognized as a trans-membrane receptor of the LGR family. In this study, we found that the level of LGR4 in Huh-7 and HepG3B cell lines was much higher than that in L02 cell lines. It indicated that the LGR4 expression was increased in HCC tissues and at cell levels. LGR4 overexpression could promote the processes of proliferation, migration, and invasion. Although the study indicated that Hsa_circ_0003945 could regulate the miR-34c-5p/LGR4/*β*-catenin axis to promote the progression of hepatocellular carcinoma [16], the exact role of LGR4 is still unclear. Mechanistically, LGR4 can activate wnt/*β*-catenin signaling in many diseases [16–18]. So, we detected the expression of catenin through immunofluorescence. It indicated that LGR4 overexpression could promote the catenin expression in the nucleus. Metformin treatment could inhibit the HCC tumorigenesis caused by LGR4 and also reduce the expression of *β*-catenin.

Alternative splicing could produce multiple transcripts of mRNA to regulate the gene expression [19]. Then, we investigated the mechanism of how metformin regulated the expression of LGR4 in HCC. We used GEO to analyze the alternative splicing. The result showed that the LGR4 gene was characterized by 3′UTR shortening upon metformin treatment in HCC cell lines. It indicated that metformin could reduce the level of LGR4 expression through alternative splicing. Finally, we used siRNA to knock down the isoforms to observe the phenotypic change in the metformin treatment. The results also showed that a specific isoform siRNA could promote the inhibition of tumorigenesis caused by metformin treatment, as well as *β*-catenin expression. Apart from alternative splicing, the genes also focus on sulfur metabolism pathways, and the study also indicated that sulfane sulfur metabolism could be used to treat cancers. We will also analyze sulfur metabolism in the future.

In conclusion, we demonstrated that LGR4 could promote the proliferation, migration, and invasion of HCC, which could be reduced by metformin through alternative splicing.

## Data Availability

The data used to support the findings of this study are available from the corresponding author upon reasonable request.
